# Case Report of Fibro-Adipose Vascular Anomaly (FAVA) with Activating Somatic *PIK3CA* Mutation

**DOI:** 10.1155/2022/9016497

**Published:** 2022-08-02

**Authors:** Jordan H. Driskill, Helena Hwang, Alexandra K. Callan, Dwight Oliver

**Affiliations:** ^1^Medical Scientist Training Program, The University of Texas Southwestern Medical Center, Dallas, TX 75390, USA; ^2^Department of Pathology, The University of Texas Southwestern Medical Center, Dallas, TX 75390, USA; ^3^Department of Orthopaedic Surgery, The University of Texas Southwestern Medical Center, Dallas, TX 75390, USA

## Abstract

Fibro-adipose vascular anomaly (FAVA) is a recently described complex and painful benign lesion found in young adults and the pediatric population composed of intramuscular vascular, fibrous, and adipose tissues. A previous report has identified the presence of somatic mosaic mutations in the gene for the catalytic subunit of phosphatidylinositol 3-kinase (*PIK3CA*) in cases of FAVA. Herein, we present a case of FAVA found in a 23-year-old male patient who presented with chronic wrist pain associated with a mass, and we identified an associated somatic activating mutation (H1047R) in *PIK3CA*. We briefly review the relevant literature surrounding the identification and histology of FAVA, the known mutational spectrum, downstream signaling pathways, and relevant treatment modalities. Our case highlights the association between FAVA and somatic mosaic activating *PIK3CA* mutations.

## 1. Case Presentation

### 1.1. Clinical Findings

**Figure 1:**
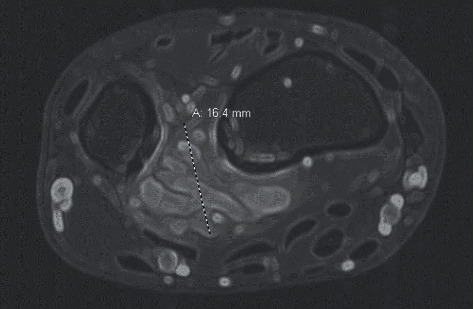
CT scan shows a lobulated 1.6 cm vascular mass in the pronator quadratus muscles along the interosseous membrane near the distal radioulnar joint.

A 23-year-old male with a complaint of right volar wrist pain on supination or pronation over the past year presented for evaluation. He denied recent trauma, although he had sustained a sports wrist injury years earlier. There was no significant family history. Physical exam was negative for a palpable mass, and bone radiologic studies were normal. At follow-up two years later, a subtle palpable wrist mass was present, and an MRI revealed a 3.4 × 1.6 × 2.5 cm multiseptated cystic volar soft tissue mass along the interosseous membrane within the pronator quadratus muscles (Figure 1). The images along with T1/T2 intensities supported the diagnosis of benign arteriovenous malformation. Biopsy of the AVM was discouraged, and the lesion was not amenable to sclerotherapy. Conservative therapy did not bring relief. Nine months later, the patient opted for surgery, during which a mass with multiple feeding vessels was found and completely resected.

**Figure 2:**
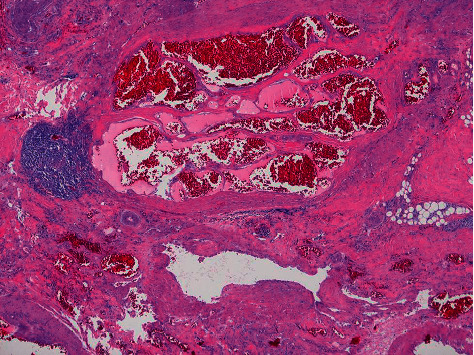
Histology (40x) shows a central cluster of thin-walled vessels, adjacent small lymphoid aggregate, focal adipose tissue, and a large vessel with a thick muscular wall.

**Figure 3:**
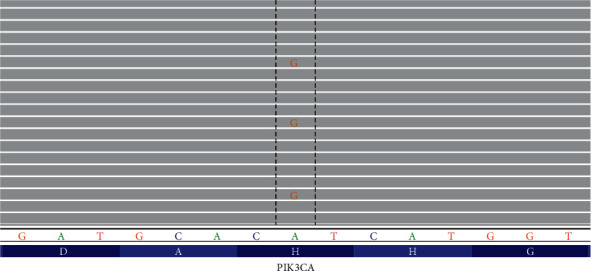
Next generation sequencing results demonstrate a low allele frequency c.3140A > G (p.His1047Arg) mutation of the PIK3CA gene. Reference amino acids and coding nucleotides are indicated at the bottom. Gray bars represent wild-type sequence.

Histologically, the tumor was an intramuscular vascular malformation composed of fibroadipose tissue, thin-walled vascular channels, abnormal large veins with muscular walls, and associated lymphoid aggregates (Figure 2). The abnormal blood vessels stained positive for CD31, and D240 highlighted focal lymphatics. PTEN immunostaining was positive (intact PTEN protein) in the vessels and in the surrounding tissue. The diagnosis given was a vascular lesion. Molecular testing was requested to determine if a mutation in the mTOR (mammalian target of rapamycin) signaling pathway was present. DNA from paraffinized sections of the vascular lesion was isolated and analyzed using a 50-gene next generation sequencing (NGS) cancer hotspot mutation assay. The only significant alteration found was a pathogenic His1047Arg mutation in the *PIK3CA* gene (Figure 3; read depth 15,576x). A low variant allele frequency indicated the mutation was somatic and not inherited through the germline. Considering the radiology, histology, and genetic findings, the diagnosis of fibroadipose vascular anomaly (FAVA) was made.

## 2. Discussion

FAVA is a rare, benign soft tissue mass most commonly found within skeletal muscle of an extremity. It was first described in 2014 with distinct clinical, radiological, and pathological features [1]. Most reported patients are pediatric females (ratio 3:1) with presenting symptoms of pain, swelling, and contractures; the most often affected site is the calf [1, 2]. Histologically, these intramuscular masses consist of nodules of thin-walled, fused, blood filled channels or sacs juxtaposed with abundant adipose and dense perivascular fibrous tissue. Smaller vein walls may be lined by an irregular smooth muscle [1, 3]. Perineural fibrosis, lymphoid aggregates, and abnormal lymphatic channels are also characteristic. Treatment historically has included splinting, sclerotherapy, cryoablation, and surgery [1, 4]. The International Society for the Study of Vascular Anomalies 2018 classification includes FAVA, along with specific venous/capillary/lymphatic malformations, CLOVES, CLAPO, Klippel–Trenaunay syndromes, as well as other disorders, as frequently associated with PIK3CA mutations [5].

Our patient was a 23 year-old male with a volar wrist mass and chronic pain recalcitrant to conservative treatment. Imaging with MRI and ultrasound suggested that the presence of a stable arteriovenous malformation was not amenable to sclerotherapy. Only after histological interpretation and molecular testing could this be identified as FAVA.

The PI3K pathway plays a critical role in normal vasculogenesis. In response to VEGF signaling, PIK3CA activates AKT, PDK1, and PKC to induce vessel formation, endothelial proliferation, and vascular permeability [6, 7]. Somatic mutations in PIK3CA have long been recognized as important cancer drivers, with approximately 80% of PIK3CA mutations clustering at E542 and E545 in the helical domain and H1047 in the catalytic domain [8, 9]. These induce constitutive kinase signaling that leads to increased activation of AKT and mTOR signaling, driving tumor cell growth and survival [10].

It may therefore be no surprise that a recent report using NGS and droplet digital PCR identified hotspot somatic mosaic *PIK3CA* mutations in 5 of 8 cases of FAVA, with mutations found at E545K, Q546K, E542K, and H1047R [11]. Interestingly, 3 of 8 patients in that study had no *PIK3CA* mutations, suggesting other mutated genes might drive vascular neoplastic formation, as noted by Hori et al. [12]. Alternatively, PIK3CA mutations affecting only a minute percent of all cells in a FAVA may not be detected by methods restricted to low sequencing depths. Importantly, the high rate of FAVA PIK3CA mutations found in the Luks, et al. series [11] supports the vascular tumor with a PIK3CA^H1047R^ mutation we report herein, also represents a FAVA. His1047Arg is the most frequently cited PIK3CA mutation in the Catalog of Somatic Mutations in Cancer (COSMIC) database [13], and early studies provided evidence that it is more oncogenic than E542K or E545K of the helical domain [14]. As the number of reported FAVA cases is low, it is difficult to ascertain whether different PIK3CA mutations correspond to different tumor behaviors.

Importance of the PI3K-AKT-mTOR axis in FAVA also comes from a study of three cases which found that mTOR activation could be observed in the abnormal blood vessels and fibrous and adipose tissues of the tumors [2]. Another case series observed that 2 of 4 patients with FAVA had H1047R mutations, and these tumors also exhibited histological evidence of activation of AKT and mTOR signaling [12]. The PI3K/AKT/mTOR signaling axis can be disrupted with inhibitors of mTOR such as rapamycin [10]. Indeed, multiple case series have identified that treatment of FAVA with rapamycin induces rapid improvements in pain and the quality of life [15, 16].

The cell type of origin of FAVA remains under investigation. As the presence of *PIK3CA* mutations leads to the activation of mTOR in the abnormal blood vessels of FAVA and also the adjacent fibrous and fatty tissues, cell-autonomous and nonautonomous effects are likely at play [2]. Similarly, it remains unknown why there is a large spectrum of overgrowth anomalies that are associated with *PIK3CA* mutations. The PIK3CA-related overgrowth spectrum (PROS) categorizes a variety of pediatric disorders, of which FAVA is now included [17], and *PIK3CA* mutations have been recently found in other vascular and lymphatic diseases such as cerebral cavernous brain malformations (CCMs) [18] and generalized lymphatic anomaly (GLA) [19]. It has been hypothesized that the difference in patient presentations of PROS is dependent on the identity and timepoint of the *PIK3CA* mutant cell that arises during embryogenesis [11].

Future studies may unravel whether somatic activating *PIK3CA* mutations alone are sufficient to lead to the development of FAVA. Mice expressing whole-body or mesodermal-restricted activated PIK3CA develop PROS, showing vascular malformations and proliferation in many different organs [20, 21]. Furthermore, lineage-restricted expression of activated PIK3CA in lymphatic endothelial cells of mice is sufficient to lead to generalized lymphatic anomaly [19], and in brain pericytes, it leads to CCMs [18]. Studies showed that expression of activated AKT, a downstream effector of PI3K, in mouse endothelial cells is sufficient to lead to aberrant angiogenesis and vascular malformations [22, 23]. Therefore, it is likely that angiogenesis induced by *PIK3CA* activation in endothelial cells is a strong contributor to the phenotype of FAVA, but additional lineage-restricted PIK3CA or AKT activation in mice, especially in muscle endothelial cells, may be demonstrative. Furthermore, treatment of FAVA with PIK3CA inhibitors, as has been done with PROS disease CLOVES [20], is an intriguing further line of inquiry.

## 3. Conclusions

FAVA is a rare, recently reported PIK3CA-related overgrowth syndrome that presents with vascular malformations and fibrofatty muscular infiltration found in the extremities of young patients. We report an additional case of a young male patient with FAVA and concurrent identification of an activating somatic *PIK3CA*^H1047R^ mutation, further establishing FAVA as a PROS disorder. Future scientific studies restricting the expression of PIK3CA to intramuscular mouse tissues may definitively establish the cell type of origin of FAVA. With known PI3K/AKT/mTOR activation in affected tissues, mTOR inhibitors remain an intriguing therapeutic possibility in patients with FAVA.

## Data Availability

There are no publicly accessible data. All patient data are HIPAA protected by our institution.
